# A Low-Cost Smart Sensor Network for Catchment Monitoring

**DOI:** 10.3390/s19102278

**Published:** 2019-05-17

**Authors:** Dian Zhang, Brendan Heery, Maria O’Neil, Suzanne Little, Noel E. O’Connor, Fiona Regan

**Affiliations:** 1 Insight Centre for Data Analytics, Dublin City University, Dublin D9, Ireland; suzanne.little@dcu.ie (S.L.); noel.oconnor@dcu.ie (N.E.O.); 2 Water Institute, Dublin City University, Dublin D9, Ireland; brendanheery@gmail.com (B.H.); maria.oneill45@mail.dcu.ie (M.O.); fiona.regan@dcu.ie (F.R.)

**Keywords:** smart sensing, water level monitoring, catchment monitoring, low-cost

## Abstract

Understanding hydrological processes in large, open areas, such as catchments, and further modelling these processes are still open research questions. The system proposed in this work provides an automatic end-to-end pipeline from data collection to information extraction that can potentially assist hydrologists to better understand the hydrological processes using a data-driven approach. In this work, the performance of a low-cost off-the-shelf self contained sensor unit, which was originally designed and used to monitor liquid levels, such as AdBlue, fuel, lubricants etc., in a sealed tank environment, is first examined. This process validates that the sensor does provide accurate water level information for open water level monitoring tasks. Utilising the dataset collected from eight sensor units, an end-to-end pipeline of automating the data collection, data processing and information extraction processes is proposed. Within the pipeline, a data-driven anomaly detection method that automatically extracts rapid changes in measurement trends at a catchment scale. The lag-time of the test site (Dodder catchment Dublin, Ireland) is also analyzed. Subsequently, the water level response in the catchment due to storm events during the 27 month deployment period is illustrated. To support reproducible and collaborative research, the collected dataset and the source code of this work will be publicly available for research purposes.

## 1. Introduction

Understanding the hydrological processes in large, open areas, such as catchments, and further modelling these processes are still open research questions. In [1], the author discussed the issues with the invalidation of computer models from environmental science perspective. In [2], Teng et al., reviewed state-of-the-art methods for flood monitoring and hydrodynamic models and concluded that no “perfect model” exists. There are still unanswered research questions that warrant addressing. The author also states that recent improvements in remote sensing and that the availability of data plays a key role in the development of new models as well as improving the accuracy of existing models. Many advanced sensing and modelling techniques have been developed in the past decades but we are still far from fully interpreting the nature of hydrological processes and we cannot measure everything that affects the hydrological environment. In fact, only a fixed range of measurements in time and space can be obtained through a limited range of techniques [3]. Traditionally, hydrologists have focused on building physical models. Building such a model, especially a generic model that can be easily transferred from catchment to catchment, is extremely challenging. Significant research effort has been devoted to quantify the response to water inputs [4,5,6,7,8].

In recent years, many researchers have switched from this traditional perceptual-based practice to statistical-based approaches, also known as the data-driven approach [9,10,11]. It has been acknowledged that field data still holds the key to hydrological environmental understanding [3]. It has been also noted that globally there is a shortage of hydrometric data and that this is hampering water management efforts [12,13]. The lack of data can be attributed to the high cost of ownership of hydrometric sensing equipment and global restrictions on funding. Significant reduction in metering density since the 1980s has been observed in Canada and US [14]. This has been recognized by government bodies, such as EU (EU data portal: https://www.europeandataportal.eu/en/), the US (U.S. Government’s open data: https://www.data.gov/) and the Irish (Ireland’s open data portal: https://gata.gov.ie) governments, who have started releasing official data to the public. However, public data is often sparse (both at temporal and spatial scales), which may not provide adequate information at the regional level. This is mainly due to the high cost of deploying monitoring stations and data collection procedures. In contrast, building, evaluating and testing hydrological models typically require years of high frequency datasets.

Recent advances in smart sensors, wireless data communications, cloud computing and machine learning have pointed hydrological research in a new direction. Low power, self-contained sensor units have been released, new wireless communication methods have been standardized, e.g., LPWAN, 4G and 5G, and fast growing cloud computing services have become available. New smart sensor network architectures for environmental monitoring tasks have been proposed [15,16]. One typical architecture of a future smart sensor network for environmental monitoring consists of three virtual layers: (1) a physical layer, where all the smart sensors reside and data pre-processing occurs, either on the sensor itself or on a field gateway; (2) the data transmission layer, where data and instruction exchange occur; (3) a data processing layer, where meaningful information is extracted and organized.

There are many definitions of “smart sensors” [17,18,19]. However, to summarize all these definitions, a smart sensor must be *intelligent* and *adaptable*. In future large-scale sensor networks, e.g., a rapidly sampling hydrometric network, the data collected will probably be far too large for traditional applications to send, store or process. Not to mention, most of the measurements carry negligible information as in most environmental monitoring networks, “normal” behaviour is not particularly interesting to researchers. Thus, a sensor unit must be intelligent and pre-process data locally on board or this process may also occur on field gateway devices depending on the sensor network structure. In this way, only condensed useful information is uploaded to the server. Also, the sensor must adapt itself to handle variations in its environment. For instance, the sensor will update its on-board data model automatically if the physical condition of the river channel changes. Another example is that the sensor may adjust the threshold associated with it when measurements start getting noisy, e.g., after a heavy rainfall or a storm event.

**Figure 1:**
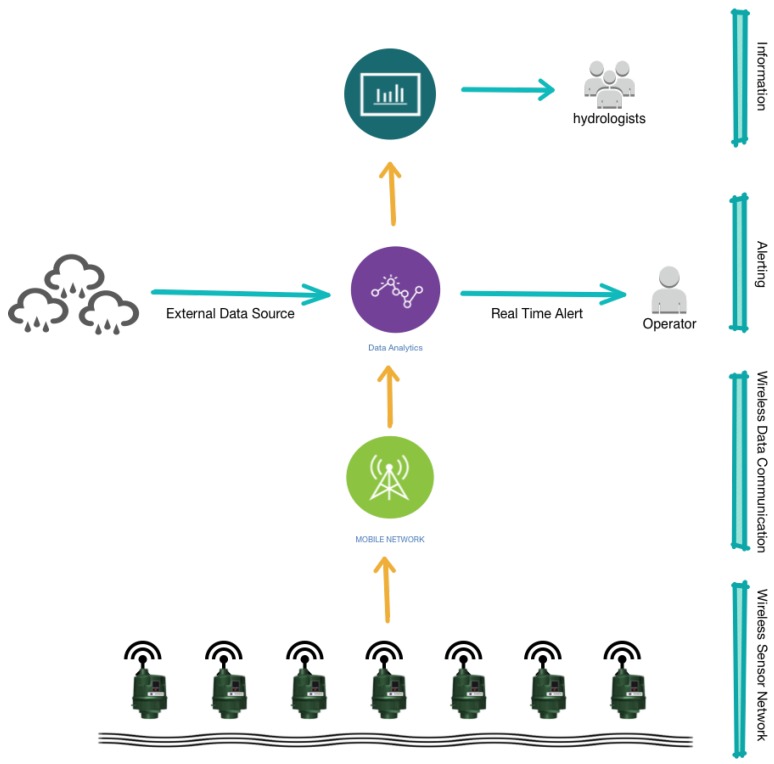
The architecture of the proposed end-to-end pipeline of catchment monitoring using low-cost smart sensor network.

In this work, we first provide a general overview of previous and current developments in hydrology and machine learning research domains and their limitations. Subsequently, the catchment used and the sensors deployed for this study is described. The performance of the deployed off-the-shelf sensor units are evaluated against the high precision and high-cost reference stations operated by South Dublin Council. These affordable sensor units provide an opportunity of bridging the gap in the availability of fine-grained “big” datasets and data-driven models. In addition, a data-driven smart sensing method MoPBAS [20] (which would potentially be built into the sensing units), is illustrated and its strengths and weaknesses are discussed as an introduction to the smart sensing concept. Moreover, events have also been constructed from the data based on the anomalies detected by the MoPBAS methods and further mapped to storm events that occurred during the deployment period at the catchment. Finally, the lag time of the River Dodder is also analyzed. To achieve this, we proposed and evaluated an automated end-to-end pipeline (as shown in Figure 1) from data capturing to information extraction processes.

The main contributions of this work are: (1) establishing the performance of an off-the-shelf, low-cost, liquid level sensor, designed for operation in a closed tank environment when deployed in an open water environment and (2) using the collected data to show a full, automated, pipe-line of data collection, data processing and information extraction processes in a catchment area. This can significantly reduce the costs of deploying a water level monitoring system at high spatial and temporal scale and potentially assist hydrologists in better understanding and managing catchments. To support reproducible and collaborative research, the collected dataset and the source code of this work is publicly available at https://github.com/DianZhang/WaterLevelMonitoring (for research purposes only).

The remainder of the paper is organized as follows: Section 2 presents an overview of the previous and current development in hydrology and machine learning. Section 3 describes the test site in this work followed by the description of the sensors deployed and the data captured. A computationally inexpensive smart sensing method is described in conjunction with fixed threshold alerting, which has been built into the sensor. The discussion of the results obtained is carried out in Section 5. Finally, Section 6 contains the conclusions and future work.

## 2. Literature Review

The cost of simple telemetered river gauges in Ireland has been estimated at up to €15,000 per installation and up to €5000 per annum for ongoing operation and maintenance [21]. Similar estimations have been reported in the USA [22]. Low-cost alternatives based on low-power Wireless Sensor Networks (WSNs) with commercial off-the-shelf sensors have been developed and tested in locations including Sao Paulo Brazil [23], the Sierra Nevada Mountains [24] and the Upper Hudson River, New York [25]. These studies were mainly focused on addressing the power and communication issues around distributed hydrometric monitoring that typically require fixed grid power supplies or solar power installations and have yet to be commercially available. The Kingspan Watchman Anywhere is a complete off-the-shelf solution, proven in the field of tank level monitoring. It is a simple and robust ultrasonic sensor with integrated battery (4 × LR14 Alkaline C batteries) and tri-band GSM/GPRS telemetry. The cost of the sensors is approximately €180 (includes VAT and delivery) per unit (Price from: https://heatingpartswarehouse.co.uk/product/watchman-anywhere-sonic-oil-level-monitor/?gclid=EAIaIQobChMIysCugebp4QIVaL7tCh1BWAicEAYYAiABEgJSFvD_BwE last accessed: 11 April 2019). The sensor unit has one year free data communication subscription and €30 per sensor per year after.

It can be installed quickly and easily, and for the duration of the field test in this work (27 months), no maintenance was required. With smart communication management and power-saving, the operational life can extend to 5 years. When adapted to river level monitoring, this offers a low-cost alternative to traditional hydrometric stations and allows for the cost effective installation of high spatial density networks.

A catchment is defined as a specific segment of the earth’s surface, set off from adjacent segments by a more or less clearly defined boundary, and occupied at any given time by a particular grouping of plants and animals [26]. Traditional hydrological models at a catchment are physically based (also known as process-based) that can be dated back to the 1960s [27]. Many approaches have been proposed since [28,29,30,31]. However, the initial optimism of physically based methods has also been challenged by the scientific community [32,33,34,35,36]. It has been argued that there are fundamental problems in the application of physically based models for practical prediction in hydrology and these problems result from limitations of the model equations relative to a heterogeneous reality. Today, understanding and modelling the hydrological processes in large areas are still open research questions. Many models have been proposed and evaluated. One of the most widely applied models is the SWAT (soil and water assessment tool model) [37,38]. It is a comprehensive model and the development of the model is still ongoing. However, the model requires a diversity of information in order to produce outputs. Furthermore, significant effort is required to configure, calibrate, run and evaluate a SWAT model. For example, the input/output document for SWAT 2009 model has over 600 pages (39 chapters) (https://swat.tamu.edu/media/19754/swat-io-2009.pdf last accessed: 20 April 2019). Other hydrological models such as MIKE SHE [29] (and its variations [39]), FEFLOW [40], MODFLOW [41] and HydroGeoSphere [42], are also applied in the literature. However, similar to SWAT, these models require extensive data and input parameters, which are sometimes not available. This makes it difficult to calibrate a model and often results in inefficient outputs. Also, both meteorological data and soil properties have a large influence on the performance of these models. A proper knowledge of subsurface flow pathways and hydraulic characteristics is necessary, otherwise, an ineffective calibrated model may perform poorly [43]. Ref. [44] provided an excellent review of current development as well as challenges in hydrology. The author also gave his vision of future trends in hydrology modelling.

On the other hand, recent developments in IoT [45,46] and cloud computing technologies [47,48] have enabled the opportunity of automated data capturing, transmitting and processing on a massive scale. Microsoft Azure, IBM Watson, SAP Leonardo, Amazon AWS etc., all provide IoT platforms as a service (PaaS) for IoT applications. This also enables the possibility of rapid development of the back-end for smart remote monitoring systems. In terms of data analysis, recent advances in machine learning, especially deep learning, have achieved near human performance in applications such as object detection in images [49,50], image caption [51,52] and machine translation [53,54]. However, these domain success models can hardly adapt to catchment modelling due to its complexity, spatial heterogeneity and lack of data. These deep models contain millions of parameters (e.g., AlexNet: 63 million [49], VGG16: 138 million [55]), which require a massive dataset to train and evaluate (AlexNet and VGG16 are trained using the ImageNet dataset, which contains over 14 million annotated images [56]). Collecting a dataset at such a scale for catchment monitoring is not feasible.

In contrast, many researchers in the environmental science domain are still focusing on developing new sensors to measure physical properties [57,58,59,60] or bio-chemical properties [61,62] of a water body. Much research has also been carried out from the catchment management perspective. Rather than build complex models and simulations, simple real, or near-real, time data-driven monitoring systems from key locations have been proposed and evaluated [63,64,65]. Research is also focused on the fusion of multiple sensing modalities that combines information from various data sources that complements each other to provide higher level information for further analysis and decision support [66].

## 3. The Deployment Site

**Figure 2:**
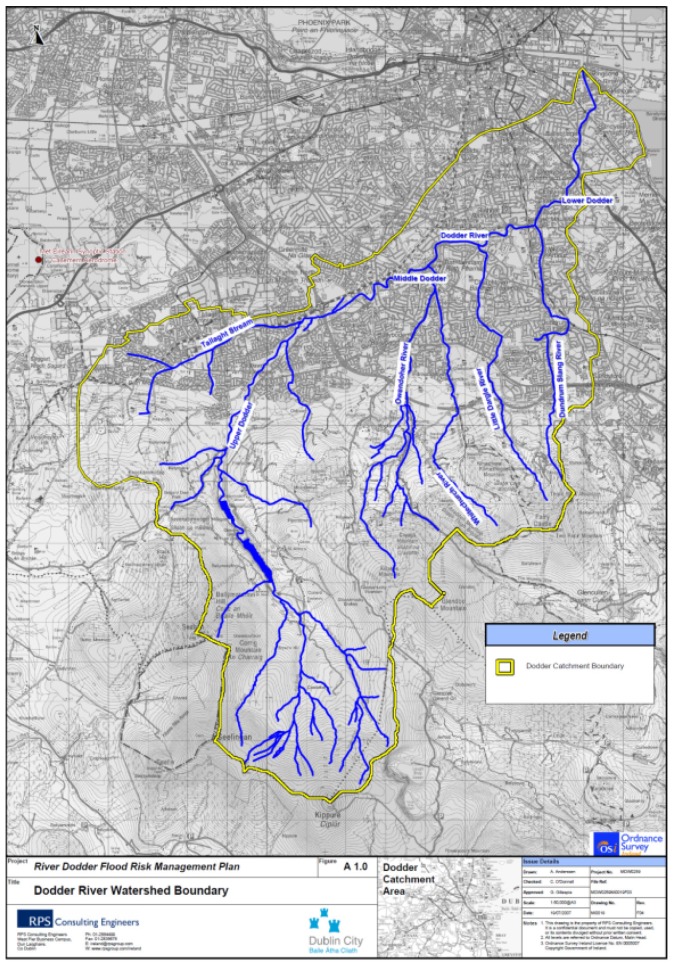
River Dodder Catchment (source: River Dodder Flood Risk Management Plan, Dublin City Council).

The Dodder river originates in the Dublin mountains to the south of Dublin City, flowing through the towns of Churchtown and Dundrum before joining the River Liffey at Dublin port and then entering Dublin Bay. The area of the catchment is 142.4 km${}^{2}$ [67]. The Dodder has five major tributaries, including the Tallaght Stream, the Owendoher stream, the Whitechurch stream, the Little Dargle and the Dundrum Slang, which contribute almost 50% of the flow. Upstream from the confluence with the Tallaght stream, there are two storage reservoirs (Glenasmole Reservoir Uppper and Glenasmole Reservoir Lower) which hold 1.6 and 0.73 million m${}^{3}$, respectively. The larger reservoir is used to supply drinking water to County Dublin, while the smaller one is used to maintain a minimum flow in the Dodder river. In advance of a heavy rainfall, the water level of the lower reservoir is reduced to provide storage capacity. The catchment, especially the lower Dodder, is known for risk of flooding due to its large change in elevation over a short distance (160 m at the lower reservoir to sea level over 13.5 km measured in a direct line). An overview of the area of the catchment is shown in Figure 2. Three major flood events have been recorded in Aug 1986, Feb 2002 and Oct 2011 with 369, 621 and 335 dwellings reported flooded in the catchment. Thus, due to the economic importance and high risk, the Dodder Catchment was selected as a suitable test site for this study.

## 4. Sensor Deployment

**Figure 3:**
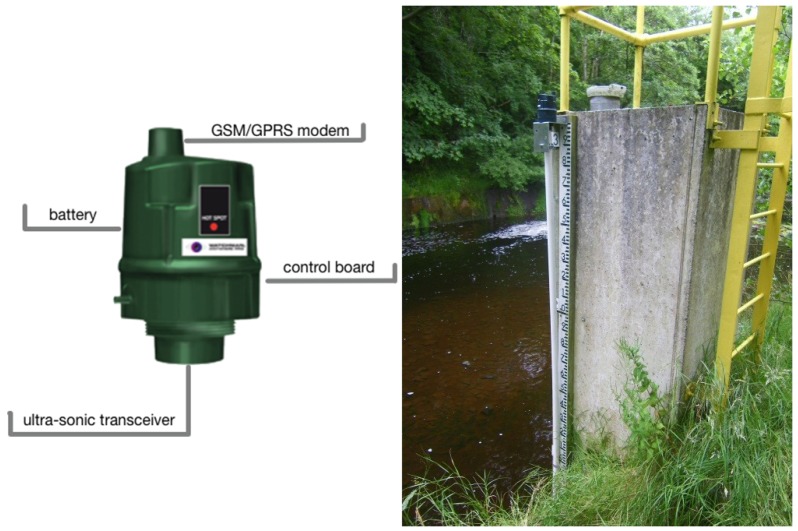
Kingspan Sonic SignalMan ultra-sonic sensor (**left**) and the sensor deployment at the Bohernabreena site (**right**).

The sensors deployed in this case study were off-the-shelf Kingspan Watchman Anywhere Pro ultra-sonic sensors that are designed for monitoring liquid levels in tanks. Liquids, such as diesel, AdBlue, lubricant additives etc., have been monitored successfully using these sensors for periods of over 5 years. The relatively low-cost (almost 100 times cheaper compared to the reference station operated by Dublin City Council that cost over €15k to construct and approximately €5k to operate and maintain annually as stated in Section 2) enables the monitoring of a catchment at a much higher spatial density than feasible with traditional, more expensive, monitoring stations. The sensor unit is capable of two-way communication that allows measurements to be sent to a cloud server and the unit to receive instructions remotely. The sensor unit itself consists of an ultrasonic transducer, tri-band GSM/GPRS module (LPWAN and 5G in future version according to Kingspan’s sensor development division), four type C LR14 Alkaline batteries, a control board and a UV Stabilized Polypropylene housing (as shown in Figure 3 left). The full specificatin of the sensor unit can be found here (https://www.kingspan.com/irl/en-ie/product-groups/service-and-telemetry/telemetry/commercial-level-monitoring/watchman-anywhere-pro last accessed 24 April 2019). A sample installation is shown in Figure 3 right, where the sensor unit is screwed into the wall sitting on top of a stilling tube, which calms the water surface and prevents interference in the signal path from intruders, such as spiders. An ultra-sonic signal is emitted, then reflected as an echo signal from the water surface before being captured by the receiver. The time interval is used by the sensor to calculate the distance to the water surface, which is converted to water level by subtracting from the distance between the sensor and the river bottom (measured during installation).

In comparison with traditional monitoring stations, the Kingspan solution has several advantages:

- Easy to deploy, 30-minute average install time.
- Easy to upgrade, remote firmware upgrade or settings update.
- No mains electric power required – safety and civil cost benefits.
- Field proven, over 30 thousand units installed for fluid level monitoring in 24 countries globally
- Built-in alert when rapid changes are detected.
- No additional maintenance required.

**Figure 4:**
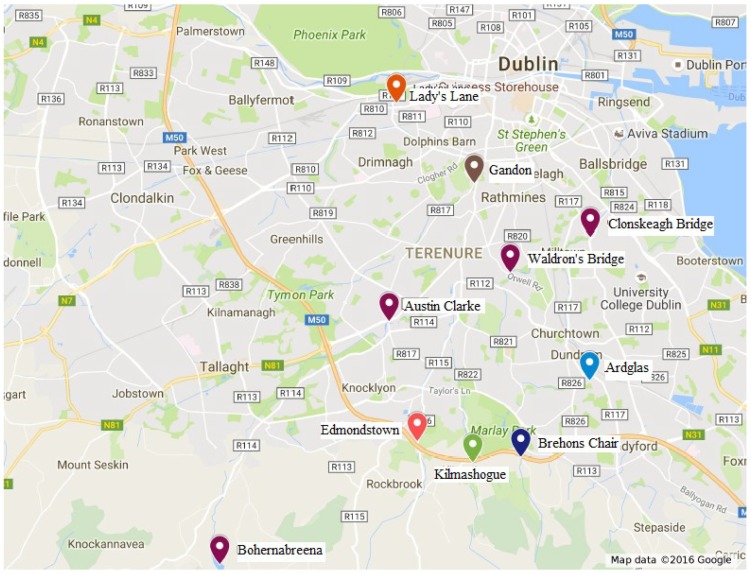
Locations of the ten deployed sensors on Goggle map.

A total of 11 sensor units were originally deployed in the South Dublin region. The geolocations of the installations are shown in Figure 4. Nine of these units were along the River Dodder and its tributaries. One unit was vandalized immediately after installation thus its location is not shown. The Bohernabreena and the Clonskeagh bridge units on the River Dodder and two further units (Gandon Close on the Poddle River and Lady’s Lane on the Camac River) were co-located with existing hydrometric stations belonging to Dublin City Council (DCC) in order to validate the performance of the sensors. Detailed deployment site information is listed in Table 1. Unfortunately, units at Brehons Chair and Edmondstown were vandalized a few months after being installed, thus, the dataset collected from these units is not included in this study.

### Data Captured

**Figure 5:**
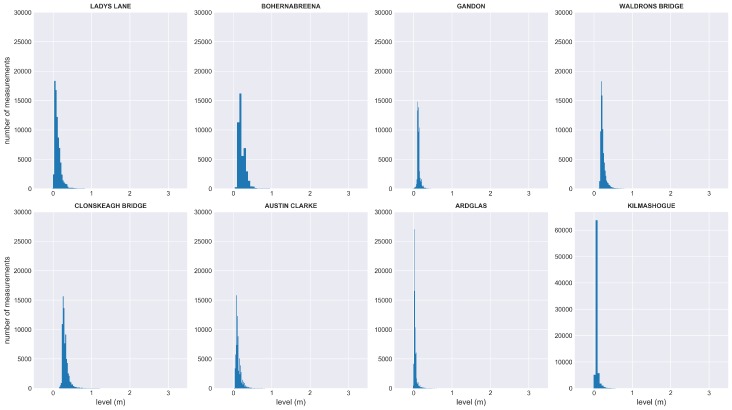
Distribution of all valid measurements from eight sensor units. Invalid values from Bohernabreena site are not included.

Originally, the sensors were set to take hourly readings. However, after comparing with the DCC reference stations, it was found that the measurements did not capture rapid variations at multiple sites. Therefore, the sampling rates of all the sensors were switched to 15 mins on 17 November 2015 by sending instructions remotely from the central control server. To optimize the battery life, the sensor stored the measurements and sent one data package every four hours unless the built-in alert level was breached, in which situation, the sensor sent the data immediately. During the 27 month deployment (since the increase of the sampling rate), a total of 624,276 readings have been received from the eight sensor units, while 1716 values were lost. A summary of the dataset is described in Table 2. The distributions of all sensor readings are shown in Figure 5.

## 5. Smart Sensing

The sensor has a built in fixed dangerous level alerting mechanism. Three dangerous levels were set individually for each of the sensor units. An alert message, green, amber or red, was sent to the corresponding operator when any of the thresholds were breached. Until 8 February 2018, a total number of 115 alerts have been received since the deployment.

To convert the units to smart sensors, we investigated a data-driven anomaly detection method. To prove the concept, the data-driven MoPBAS [20] anomaly detection was applied to the captured data. As described in [20], the method is specifically designed for environmental sensors. There are several advantages including:

- Low hardware requirement (the algorithm can be built into the sensor)
- Computationally inexpensive (anomalies can be detected in real time)
- A compositional small training data set is required (model can be built as soon as a small set of data, e.g., 50 readings, has been received)
- Easy to tune (initial parameters can be set based on site survey)
- Dynamic modeling (model is trained based on the data captured by the sensor. Thus, every model is trained based on the variation of the measurements at a site)
- Dynamic updating (model is updated in real-time when new data arrives)
- Dynamic threshold (detection threshold is constantly updating based on variation of the measurements)

Once the anomalies are detected, they are grouped into events based on their temporal information. Contiguous anomalies are considered as the same event.

## 6. Results and Discussion

### 6.1. Deployment Issues

Throughout the duration of the deployment, there was no field maintenance required. This combined with the low-cost of installation meant that the total cost of ownership of a sensor was significantly lower than traditional monitoring stations. In addition, the sensors were self-powered, which means that they could be installed “anywhere” along a water channel without the constraint of being close to a power source.

However, during our test period, two technical issues were encountered, signal loss and invalid readings. 1716 (0.275%) measurements did not reach the data server, as shown in Table 2. Consulting with Kingspan engineers, this was mainly due to faulty mobile network connections. However, this issue could be easily solved through a firmware update. A handshaking system using the sensor’s two-way data communication capability could be implemented where the server can request the missing values after an expected package of data is lost. The only requirement for this solution is that the sensor needs to buffer the data for a short period (until the server sends a confirmation or after a pre-defined time interval has past). The second problem experienced is the invalid data received from the Bohernabreena sensor unit after 6 March 2016. Although the sensor unit is still “functioning”, the measurements received are completely random. On-site inspection found that spiders climb into the tube during the dry period when the bottom of the tube is exposed. Spiders started nesting inside the tube since it is warmer. The spider webs reflect the ultra-sonic signal in a random pattern resulting in random values measured by the sensor’s receiver. The sensor unit has been brought back to the lab and tests show that the unit itself is functioning properly.

**Figure 6:**
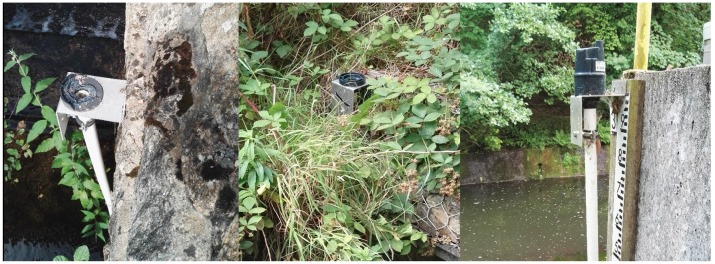
Edmondstown sensor unit (**left**) is removed by brute force. Base bracket is broken. Brehons Chair unit (**middle**) is unscrewed. Base bracket is intact. Both of these units are clearly visible from a public road and easy to reach. Spider webs were found in the tube of the Bohernabreena sensor unit (**right**), which caused invalid readings.

Two units that are deployed at Brehons Chair and Edmondstown were vandalized; one removed by brute force and the other removed from its mountings. Both of the sensor units were clearly visible and easy to reach. Photos taken from site inspection are shown in Figure 6.

### 6.2. Sensor Performance

**Figure 7:**
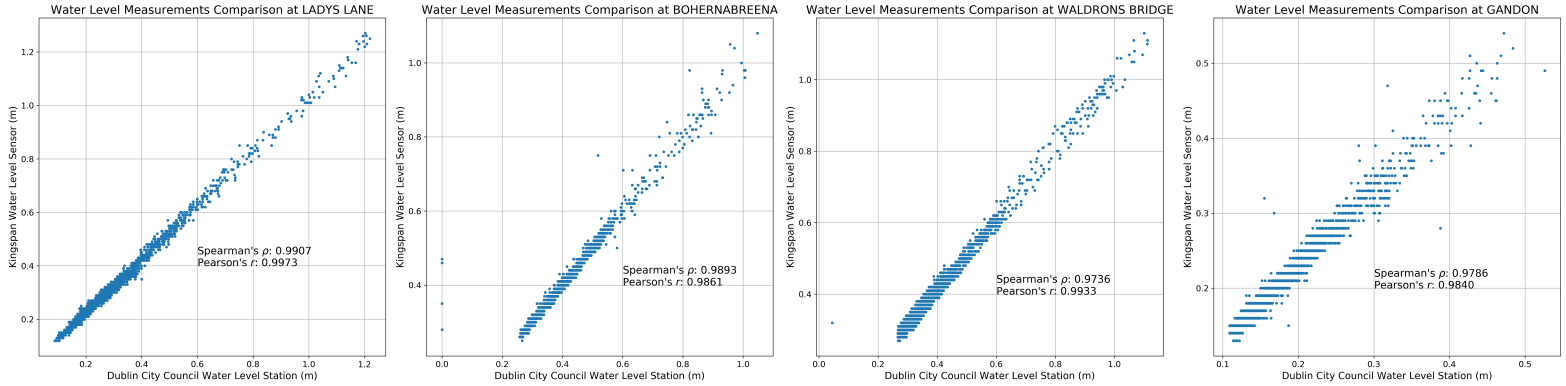
Correlation between the four Dublin City Council reference stations and Kingspan sensor units deployed.

Since the sensor was originally designed for monitoring liquid levels in a sealed tank, it is essential to examine its accuracy in an open environment and to confirm that the sensor is suitable for catchment monitoring tasks. To validate the performance of the deployed sensor, data were analyzed from the four units, which were installed alongside the DCC hydrometric stations. To compare the readings, Pearson correlation and Spearman’s rank correlation were used. Both methods are commonly used to identify the relationship between two variables. The key difference between the two methods is that Spearman’s rank correlation can identify non-linear relationships. The correlation coefficients as well as the mean absolute error (MAE) are shown in Table 3 (using one month data, Dec 2015, provided by Dublin City Council). Both of the 95% and 99% confidence intervals are listed in Table 3. As can be seen from the table, within 99 percent of the time, the sensor measurements have less than 5 mm difference compared with readings from the reference stations. A comparison between the DCC stations and the Kingspan units of the four sites is shown in Figure 7. As can be seen from the graph, the two datasets do have a strong positive relationship (with some noise). The results show that the the water levels measured by Kingspan units are very close to the DCC readings. This illustrates that the Kingspan units provide almost identical water level information with much lower costs.

As can been seen from the results, the low-cost remote sensor measurements are very close to the reference DCC stations. This means that the water level at a catchment can be monitored with high precision at a much more affordable price. Also, due to the simple installation procedure, far more locations on a water body can be monitored, providing richer information when understanding the response of a catchment.

### 6.3. Real-Time Alerts

Since the deployment, a total of 115 valid alerts were raised by the sensors. As described, each sensor unit had three pre-defined alert levels. When any of these levels were breached, the sensor unit sent the data to the server immediately and the server issued a warning message to the registered operators. This mechanism could also be used as sluice auto control, for instance, to open a water gate automatically when an alert is received and close the gate after the water level falls. A summary of the alerts is described in Table 4.

### 6.4. Anomaly Detection

The benefit of anomaly detection is that it can automatically isolate abnormal readings from the data stream that need to be further analyzed by a hydrologist. This can significantly reduce manual data filtering work especially when a large-scale sensor network is deployed. In addition, as described in [68], the anomalies can be further grouped into events based on their temporal information. More sophisticated analysis can potentially be applied, such as clustering (which finds the common variations and ultimately the causes) and classification (which assigns an event to a pre-defined class). Since the source of a water body is known (rainfall, discharge of a reservoir, surface flow, etc.), this event database may give indicators of future response. For example, we know the previous response of a catchment due to a heavy rainfall. It is expected that the catchment has a similar response to a similar future rainfall event.

**Figure 8:**
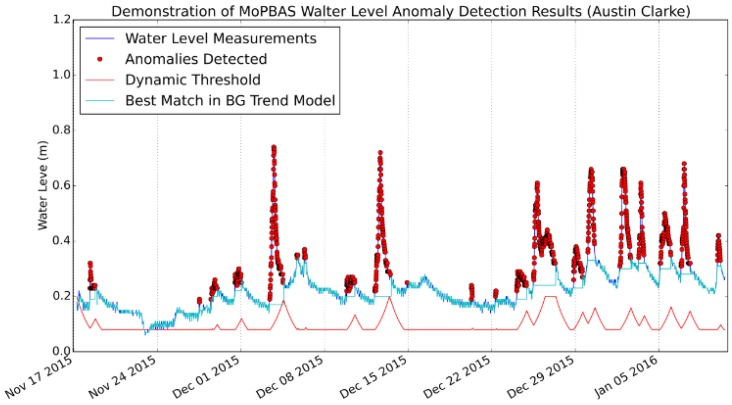
Demonstration of MoPBAS anomaly detection results at Austin Clake site with dynamic threshold and background (BG) trend model.

**Figure 9:**
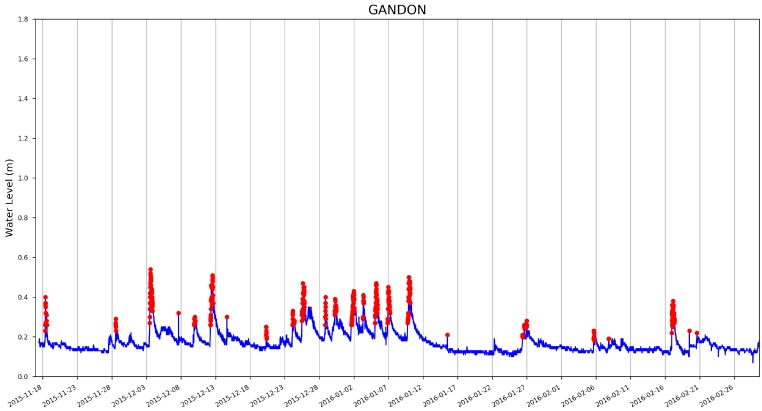
Anomaly detection results (first 10,000 measurements) using MoPBAS at Gandon site.

**Figure 10:**
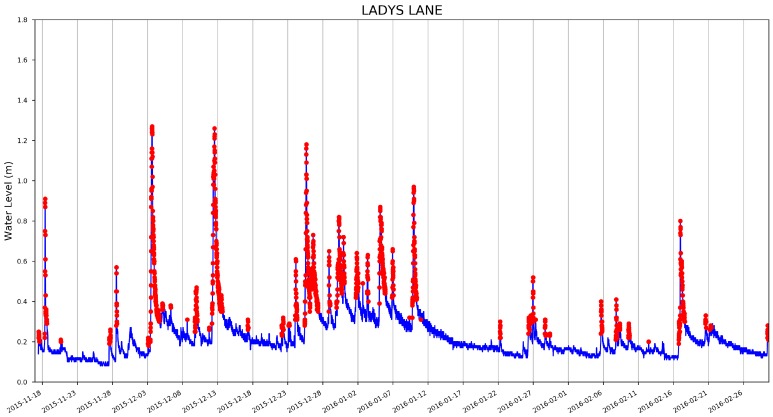
Anomaly detection results (first 10,000 measurements) using MoPBAS at Lady’s Lane site.

A sample of the anomaly detection results is shown in Figure 8 (with the dynamically updating threshold and background trend model), Figure 9 and Figure 10 (for illustration purpose, the first 10,000 samples are shown). As can be seen from the graph, rapid changes of the water level have been detected. In the context of this paper, the rapid changes are defined as significant deviation from the water level trend rather than significant difference from a pre-defined fixed value.

It can be seen in Figure 8 that although the absolute levels around 8 December 2015 are higher than the first few measurements classified as anomalies after 24 November 2015, they are still considered as “normal” since they are not significantly above the overall trend level (which has increased). Also, as illustrated in Figure 8, the dynamic threshold (red continuous line) is constantly updated and increased when the water level rises rapidly and the measurements start getting noisy. The benefit of this mechanism is that when an “event” happens, the sensor readings become noisy (this is true for many environmental monitoring tasks), thus, the increase in the threshold gives the model more tolerance to handle this noise. Also, the model is quickly updated as soon as the water level falls after an “event”. The number of anomalies detected at each site is shown in Table 5.

The initial MoPBAS anomaly detection parameters set for all the eight sites are the same and are empirically set. This, once again, shows the power of a data-driven approach in which the model and its parameters are constantly updated based on the data received. Also, a generic data-driven method and one parameter set are performing well on multiple sites, which have different characteristics. More detail of how to set these parameters, e.g., increase or decrease the sensitivity of the detection can be found in [68]. The following list shows all the hyper-parameter values used in these experiments.

- *N*: The number of elements in the trend model is set to 24.
- t_back_thresh: The initial value of dynamic background threshold is set to 0.1.
- t_upper_bound: The upper bound of the dynamic background threshold is set to 0.25.
- t_lower_bound: The lower bound of the dynamic background threshold is set to 0.1.
- t_inc_dec: The dynamic background threshold increase/decrease step is set to 0.03.
- t_scale: The background dynamic scale is set to 1.0 (no scale), enlarge the scale of background dynamic to the same range as background threshold.
- l_learning_rate: The learning rate of the background model is set to 1.0.
- l_inc: The learning rate increase step is set to 1.0.
- l_dec: The learning rate decrease step is set to 0.1.
- l_upper_bound: The learning rate upper bound is set to 1.0.
- l_lower_bound: The learning rate lower bound is set to 0.05.
- is_upward_detect: False since only rapid increases are interested.

### 6.5. Events

Based on the temporal information of the anomalies detected, consecutive anomalies are grouped together as *events*. To accommodate noise, two nearby anomalies that are separated by one normal measurement are still grouped together as in the same event. The total number of events created for all the sites is shown in Table 6. These events are further mapped to the storm events that occurred during the same period and this is discussed below.

### 6.6. Lag Time Analysis

To understand the response of the Dodder catchment, lag time analysis was carried out using the data captured from the four sensor units along the main flow path, which starts from the upper reservoir to the lower Dodder stream (the path can be seen from Figure 2 and Figure 4). The idea was to calculate the correlation between two locations along the water flow channel with an offset time window and record the number of offsets at the highest correlation.

**Figure 11:**
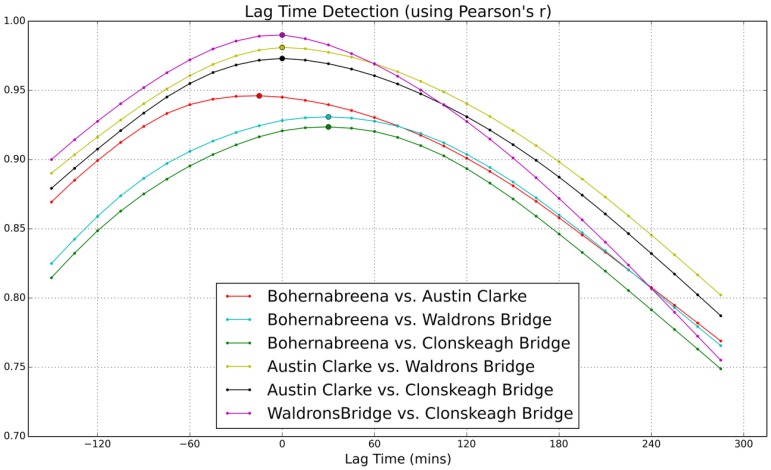
Correlation between the sites along one flow path of the Dodder catchment.

Five curves (apart from the correlation between Bohernabreena and Austin Clarke) in Figure 11 suggest that the lag time between upper stream (Bohernabreena and Austin Clarke) to downstream (Waldrons Bridge and Clonskeagh Bridge) is two offsets. Thus, the response time at the lower Dodder catchment has a 30 minute delay (one offset is one sampling interval, which is 15 min) relative to the upstream. However, the results show that the lag time between Bohernabreena and Austin Clarke is minus one offset, which means that the response from the Austin Clarke site is 15 min earlier than the upper stream Bohernabreena site. This result conflicts with the other five curves shown in Figure 11. This is most likely because of the functioning of the reservoir just upstream of the Bohernabreena sensor. The rain which fell in the downstream catchment would have entered the rivers and raised the level within a short time frame, whereas rain which fell in upper catchment would have filled up the reservoir before proceeding to downstream and influencing the river level.

**Figure 12:**
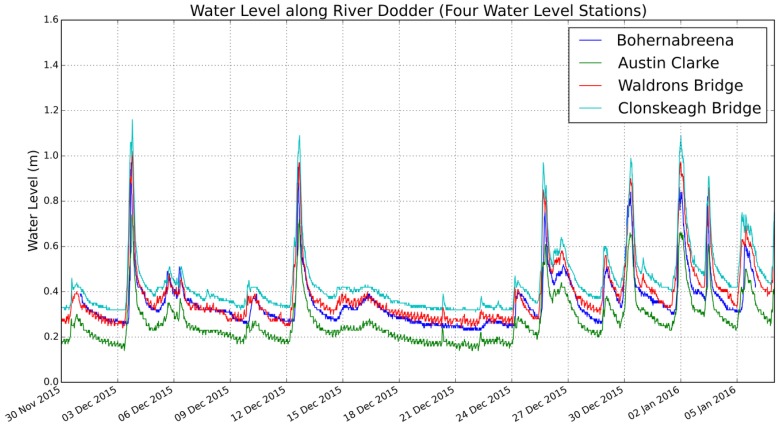
Raw sensor readings from Bohernabreena, Austin Clarke, Waldrons Bridge and Clonskeagh Bridge. Lag time can not be visually identified.

However, we can draw the conclusion that in the Dodder catchment, the lag time of the lower stream is very small compared to the upper stream. This also can be seen from Figure 12 (raw sensor data from the four sensor units), where lag time can not be visually identified.

## 7. Storms and the Water Levels

**Figure 13:**
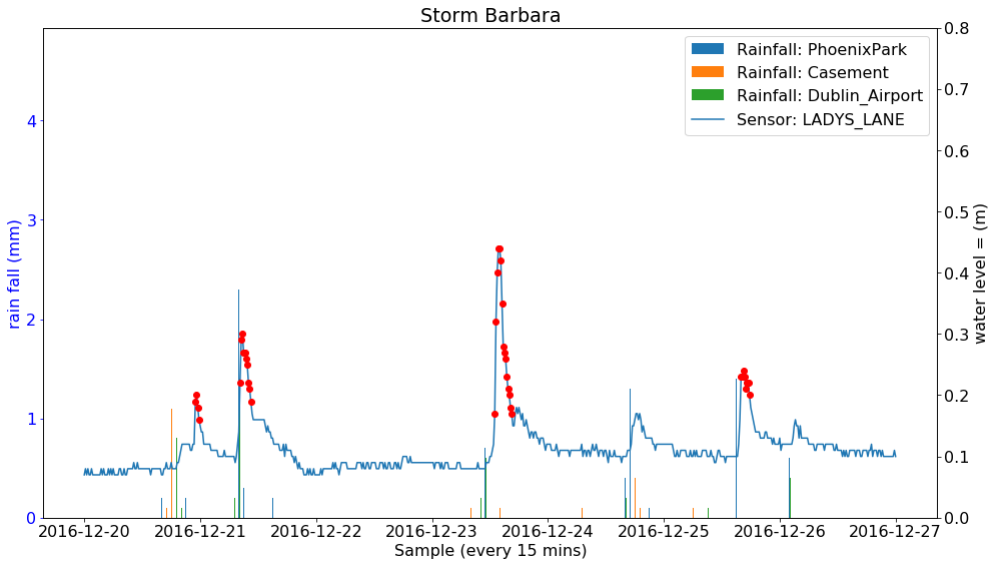
Rainfall from three Met Eireann weather stations and the water level sensor measurements along with the anomalies detected and the four events constructed during the Storm Barbara.

**Figure 14:**
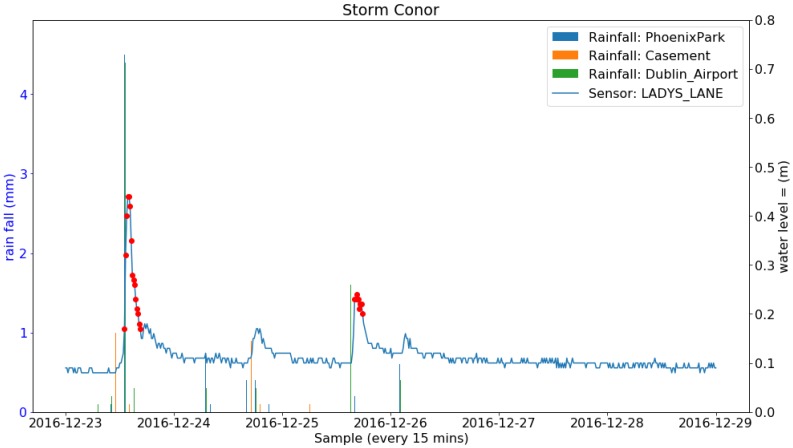
Rainfall from three Met Éireann weather stations and the water level sensor measurements along with the anomalies detected and the two events constructed during the Storm Conor.

**Figure 15:**
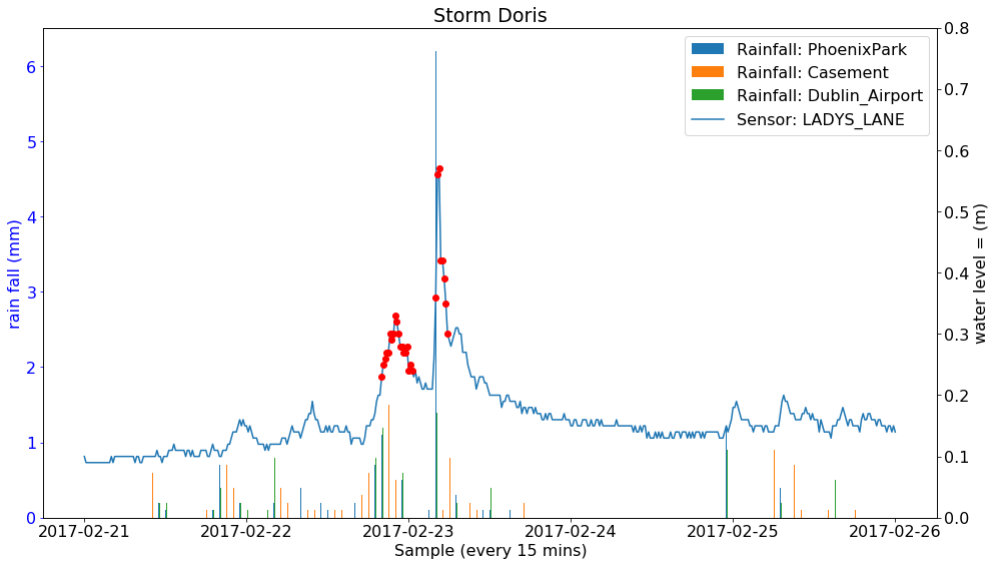
Rainfall from three Met Éireann weather stations and the water level sensor measurements along with the anomalies detected and the two events constructed during the Storm Doris.

**Figure 16:**
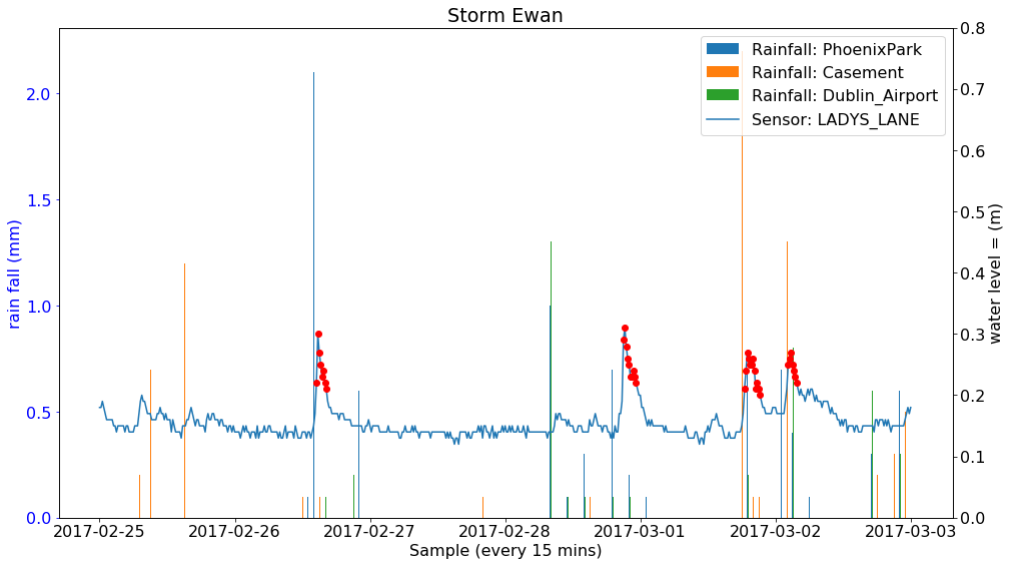
Rainfall from three Met Éireann weather stations and the water level sensor measurements along with the anomalies detected and the four events constructed during the Storm Ewan.

During the deployment period, 24 storms occurred in Ireland. A number of them brought heavy rainfall and others only strong wind. The total amount of rainfall (Rainfall data retrieved from Met Eireann at www.met.ie under a Creative Commons Attribution-Share Alike 4.0 International licence) during each of the storms from three weather stations in Dublin are listed in Table 7. Four successive and also the most rainy storms (named Barbara, Conor, Doris and Evan) and the sensor readings at the Lady’s Lane (the closest sensor to the weather stations) are shown in Figure 13, Figure 14, Figure 15 and Figure 16. It can be clearly seen that the water level at the Lady’s Lane sensor has a positive response to the heavy rainfall. In addition, it can be also seen from the graphs that all the rapid increases of the water level at the site are detected by the anomaly detection algorithm. There are many benefits of coupling anomaly detection and rainfall monitoring. The algorithm can automatically extract rapid changes of water level after rainfall events at large scales and in real time. This can be used to build early flood warning systems. Since the model is based on local historical data, it might be more accurate at local level. It can also help to build and evaluate rainfall run-off models since it can separate rapid changes from long term trends. Also, as the method is purely data-driven, the system can be fully automated.

## 8. Conclusions

In this work, we first evaluated the performance of low-cost off-the-shelf Kingspan Watchman Anywhere Pro sensors for open water level monitoring tasks. The performance is very promising, which indicates that this self-contained unit can measure water levels of an open water body almost as accurately as traditional sophisticated stations with a small fraction of the cost. This indicates that the dataset collected is valid and provided sufficient information for the subsequent analysis. In addition, the proposed full end-to-end pipeline was evaluated using the collected dataset as a case study to illustrate a fully automated data collection, transmission and information extraction system. We demonstrated a data-driven anomaly detection method that can automatically adapt based on the variation at the site. This enables the possibility of creating a large-scale data-driven smart water level sensor network that can automatically adapt based on the characteristics of the target site. As an example of the utility of the system, lag time along the River Dodder was also analyzed and the results show that there was no significant lag time along the river. The reason could be that the rapid change in water level sensor measurements was only due to heavy rainfalls, which generally covers the whole catchment. The water enters the river through surface run-off, drain systems, etc., concurrently along the whole river channel. Thus, the water levels at all the deployed locations rise almost simultaneously. Finally, the rainfall values from all major storm events during the test period were also analyzed. The results show that all the rapid changes in water levels after storm events that have heavy rainfall were successfully detected. The benefit of coupling anomaly detection and, subsequently, abnormal event construction with storm events is that it provides large-scale fine-grained water level response to heavy rainfall at a local level, which can subsequently contribute to data-driven rainfall modeling. The data-driven anomaly detection method combined with the self-contained sensor unit provided a fully automatic pipeline for this end-to-end process. The pipeline described in this work provided the opportunity of extracting high-level information at high spatial and temporal scales automatically, which can potentially further assist hydrologists to better understand the hydrological processes in large areas.

Recent developments (such as deep learning) in computer science, especially in the big data analysis domain have shown significant improvement in performance and achieved near-human performance (e.g., in image captioning, object detection in images) or exceed human performance (e.g., AlphaGO) in many applications. As future work, we will try to adopt these successful methods to the catchment monitoring domain, either by fine-tuning an existing model or retraining a model from scratch. LSTM (long-short term memory), a type of recurrent neural network (RNN), has shown excellent accuracy in predictive and time series applications, such as machine translation, and will be evaluated using the dataset collected in this work.

## Figures and Tables

**Table 1:** Details of installed sensor units along the Dodder catchment. *Bohernabreena sensor readings are not valid after 06/03/2016 due to spiders in the tube.

Site Name	Dates Active	Reference Station	River
LADY’S LANE	24/07/2015 to 08/02/2018	Yes	Camac
BOHERNABREENA	24/07/2015 to 08/02/2018 *	Yes	Dodder
GANDON	24/07/2015 to 08/02/2018	Yes	Poddle
WALDRONS BRIDGE	24/07/2015 to 08/02/2018	Yes	Dodder
CLONSKEAGH BRIDGE	24/07/2015 to 08/02/2018	No	Dodder
KILMASHOGUE	26/08/2015 to 08/02/2018	No	Whitechurch Stream
AUSTIN CLARKE	26/08/2015 to 08/02/2018	No	Dodder
ARDGLAS	26/08/2015 to 08/02/2018	No	Dundrum Slang

**Table 2:** Data received from all sensor units and the maximum, minimum and the standard deviation of the measurements. *max level reading that might be noise. ** Standard divination (std) is calculated on valid data (first 45,537 readings collected before 06/03/2016) only at Bohernabreena site.

Site	No. Reading	Reading Lost	Max Level	Min Level	std
LADY’S LANE	77,918	280	1.51	−0.01	0.094387
BOHERNABREENA	78,066	533	1.08 (2.9 *)	−0.01	0.093907 **
GANDON	77,950	248	0.59	−0.01	0.042662
WALDRONS BRIDGE	77,918	287	1.13	−0.01	0.076345
CLONSKEAGH BRIDGE	77,966	232	1.29	−0.01	0.085595
KILMASHOGUE	78,158	40	0.57 (2.58) *	−0.01	0.039698
AUSTIN CLARKE	78,142	56	0.82	−0.01	0.073746
ARDGLAS	78,158	40	0.60	−0.01	0.041509
Total	624,276	1716			

**Table 3:** Comparison results between Dublin City Council stations and the deployed sensor units using Pearson and Spearman’s rank correlation. The mean absolute error and both of the 95% and 99% confidence intervals of the measurement differences are also shown (all in millimeter).

	Lady’s Lane	Bohernabreena	Waldron’s Bridge	Gandon
Spearman’s	0.9907	0.9893	0.9736	0.9786
Pearson	0.9973	0.9861	0.9933	0.9840
MAE (mm)	0.92	0.76	1.12	0.74
95% (mm)	1.23	1.85	1.55	1.11
99% (mm)	3.18	4.76	4.00	2.87

**Table 4:** Level alerts received from the sensors. Due to the upgrading of the Kinspan app system, no alert was received between 9 January 2016 to 2 August 2016. 22 invalid alerts rose from Bohernabreena after it failed to send valid measurements are not included.

Site Name	No. Alerts
LADY’S LANE	36
BOHERNABREENA	14
GANDON	12
WALDRONS BRIDGE	24
CLONSKEAGH BRIDGE	29
KILMASHOGUE	0
AUSTIN CLARKE	0
ARDGLAS	0
Total	115

**Table 5:** Total number of anomalies detected using MoPBAS.

Site Name	No. Anomalies Detected
LADY’S LANE	3790
BOHERNABREENA	2123
GANDON	1286
WALDRONS BRIDGE	3179
CLONSKEAGH BRIDGE	4206
KILMASHOGUE	430
AUSTIN CLARKE	1930
ARDGLAS	945
Total	17,889

**Table 6:** Total number of events grouped based on the temporal information of the anomalies detected.

Site Name	No. Events
LADY’S LANE	240
BOHERNABREENA	63
GANDON	106
WALDRONS BRIDGE	131
CLONSKEAGH BRIDGE	128
KILMASHOGUE	25
AUSTIN CLARKE	81
ARDGLAS	104
Total	878

**Table 7:** Storms occurred during the deployment period and the total rainfall from three weather stations in Dublin.

Storm	Time	Casement (mm)	Airport (mm)	Phoenix Park (mm)
Abigail	12–13/11/2015	4	7.1	5
Barney	17–18/11/2015	3.6	5.8	3.4
Clodagh	29/11/2015	0	0	0
Desmond	4–6/12/2015	12	14.6	11.5
Eva	23–24/12/ 2015	4	1.3	3.9
Frank	29–30/12/ 2015	6	3	4
Gertrude	29/01/2016	0	0.1	0
Henry	1–2/02/2016	2.3	1.8	1.7
Imogen	7–8/02/2016	3.5	2	3.5
Jake	2/03/2016	0	0	0
Katie	28/03/2016	0	0	0
Angus	19–22/11/2016	1.9	2.6	1.7
Barbara	20–27/12/2016	17.6	14.2	15.8
Conor	23–29/12/2016	11	10.2	10.9
Doris	21–26/02/2017	24.4	15.5	22.1
Ewan	25/022017–03/03/2017	19.3	13.1	17.9
Aileen	12–13/09/2017	6.6	7.2	7.1
Brian	21/10/2017	0	0	0
Caroline	7–10/12/2017	6.5	4.4	5.6
Dylan	30–31/12/2017	6.8	13.9	11.4
Eleanor	2–3/01/2018	8.5	8.4	6.2
Fionn	16/01/2018	0	0	0
Georgina	23–24/01/2018	0.2	0	0.4

## References

[1] Beven K., Lane S. (2019). Invalidation of Models and Fitness-for-Purpose: A Rejectionist Approach. Computer Simulation Validation.

[2] Teng J., Jakeman A.J., Vaze J., Croke B.F., Dutta D., Kim S. (2017). Flood inundation modelling: A review of methods, recent advances and uncertainty analysis. Environ. Modell. Softw..

[3] Beven K.J. (2011). Rainfall-Runoff Modelling: The Primer.

[4] Linden D., Dixon R. (1973). Infiltration and Water Table Effects of Soil Air Pressure Under Border Irrigation 1. Soil Sci. Soc. Am. J..

[5] Ehlers W. (1975). Observations on earthworm channels and infiltration on tilled and untilled loess soil. Soil Sci..

[6] Wang Z., Feyen J., van Genuchten M.T., Nielsen D.R. (1998). Air entrapment effects on infiltration rate and flow instability. Water Resour. Res..

[7] Mishra S.K., Singh V.P. (1999). Another look at SCS-CN method. J. Hydrol. Eng..

[8] Marshall M.R., Francis O.J., Frogbrook Z.L., Jackson B.M., McIntyre N., Reynolds B., Solloway I., Wheater H.S., Chell J. (2009). The impact of upland land management on flooding: Results from an improved pasture hillslope. Hydrol. Processes.

[9] Vernieuwe H., Georgieva O., De Baets B., Pauwels V.R., Verhoest N.E., De Troch F.P. (2005). Comparison of data-driven Takagi–Sugeno models of rainfall–discharge dynamics. J. Hydrol..

[10] Yu P.S., Chen S.T., Chang I.F. (2006). Support vector regression for real-time flood stage forecasting. J. Hydrol..

[11] Prinzio M.D., Castellarin A., Toth E. (2011). Data-driven catchment classification: Application to the pub problem. Hydrol. Earth Syst. Sci..

[12] Stokstad E. (1999). Scarcity of rain, stream gages threatens forecasts. Science.

[13] Gilbert N. (2010). How to avert a global water crisis. Nature.

[14] Mishra A.K., Coulibaly P. (2009). Developments in hydrometric network design: A review. Rev. Geophys..

[15] Zhang Z., Glaser S.D., Bales R.C., Conklin M., Rice R., Marks D.G. (2017). Technical report: The design and evaluation of a basin-scale wireless sensor network for mountain hydrology. Water Resour. Res..

[16] Skalka C., Frolik J. (2014). Snowcloud: A complete data gathering system for snow hydrology research. Real-World Wireless Sensor Networks.

[17] Meijer G. (2008). Smart Sensor Systems.

[18] Hunter G.W., Stetter J.R., Hesketh P., Liu C.C. (2010). Smart sensor systems. Electrochem. Soc. Interface.

[19] Frank R. (2000). Understanding Smart Sensors.

[20] Zhang D., Sullivan T., Briciu Burghina C.C., Murphy K., McGuinness K., O’Connor N.E., Smeaton A.F., Regan F. (2014). Detection and classification of anomalous events in water quality datasets within a smart city-smart bay project. Int. J. Adv. Intell. Syst..

[21] OPW (2009). Eastern CFRAM Study - Liffey Flood Controls & Flood Forecasting System Option. https://www.opw.ie/en/media/Liffey%20Flood%20Controls%20and%20Flood%20Forecasting%20System%20Option%20report.pdf.

[22] Babbit B., Groat C.G. (1999). Streamflow Information for the Next Century—A Plan for the National Streamflow Information Program of the U.S. Geological Survey. https://apps.dtic.mil/dtic/tr/fulltext/u2/a442013.pdf.

[23] Hughes D., Ueyama J., Mendiondo E., Matthys N., Horré W., Michiels S., Huygens C., Joosen W., Man K.L., Guan S.U. (2011). A middleware platform to support river monitoring using wireless sensor networks. J. Braz. Comput. Soc..

[24] Taneja J., Jeong J., Culler D. Design, modeling, and capacity planning for micro-solar power sensor networks. Proceedings of the 7th International Conference on Information Processing in Sensor Networks.

[25] Islam M.S., Bonner J.S., Paley J.B., Fuller C.B. (2016). Low-cost stand-alone system for real-time hydrological monitoring. Environ. Eng. Sci..

[26] Cooper C.F. (1969). Chapter IX Ecosystem Models in Watershed Management. The Ecosystem Concept in Natural Resource Management.

[27] Crawford N.H., Linsley R.K. (1966). Digital Simulation in Hydrology’ Stanford Watershed Model 4.

[28] Stephenson G.R., Freeze R.A. (1974). Mathematical simulation of subsurface flow contributions to snowmelt runoff, Reynolds Creek Watershed, Idaho. Water Resour. Res..

[29] Abbott M.B., Bathurst J.C., Cunge J.A., O’Connell P.E., Rasmussen J. (1986). An introduction to the European Hydrological System—Systeme Hydrologique Europeen, “SHE”, 1: History and philosophy of a physically based, distributed modelling system. J. Hydrol..

[30] Young A. (1989). A nonpoint-source pollution model for evaluating agricultural watersheds. J. Soil Water Conserv..

[31] Hill M.C. (1992). A Computer Program (MODFLOWP) for Estimating Parameters of A Transient, Three-Dimensional Ground-Water Flow Model Using Nonlinear Regression.

[32] Beven K. (1989). Changing ideas in hydrology—The case of physically based models. J. Hydrol..

[33] Winter T.C. (1981). Uncertainties in estimating the water balance of lakes 1. J. Am. Water Resour. Assoc..

[34] Beven K.J., Cloke H.L. (2012). Comment on “Hyperresolution global land surface modeling: Meeting a grand challenge for monitoring Earth’s terrestrial water” by Eric F. Wood et al. Water Resour. Res..

[35] Beven K. (2019). Validation and Equifinality. Computer Simulation Validation.

[36] Seibert J., Staudinger M., van Meerveld H.I. (2019). Validation and Over-Parameterization—Experiences from Hydrological Modeling. Computer Simulation Validation.

[37] Douglas-Mankin K., Srinivasan R., Arnold J. (2010). Soil and Water Assessment Tool (SWAT) model: Current developments and applications. Trans. ASABE.

[38] Arnold J.G., Moriasi D.N., Gassman P.W., Abbaspour K.C., White M.J., Srinivasan R., Santhi C., Harmel R., Van Griensven A., Van Liew M.W. (2012). SWAT: Model use, calibration, and validation. Trans. ASABE.

[39] Andersen J., Refsgaard J.C., Jensen K.H. (2001). Distributed hydrological modelling of the Senegal River Basin—Model construction and validation. J. Hydrol..

[40] Trefry M.G., Muffels C. (2007). FEFLOW: A finite-element ground water flow and transport modeling tool. Groundwater.

[41] Harbaugh A.W., Banta E.R., Hill M.C., McDonald M.G. (2000). MODFLOW-2000, The U. S. Geological Survey Modular Ground-Water Model-User Guide to Modularization Concepts and the Ground-Water Flow Process. Open-File Report. U. S. Geological Survey.

[42] Brunner P., Simmons C.T. (2012). HydroGeoSphere: A fully integrated, physically based hydrological model. Groundwater.

[43] Devia G.K., Ganasri B., Dwarakish G. (2015). A Review on Hydrological Models. Aquatic Procedia.

[44] Fatichi S., Vivoni E.R., Ogden F.L., Ivanov V.Y., Mirus B., Gochis D., Downer C.W., Camporese M., Davison J.H., Ebel B. (2016). An overview of current applications, challenges, and future trends in distributed process-based models in hydrology. J. Hydrol..

[45] Madakam S., Ramaswamy R., Tripathi S. (2015). Internet of Things (IoT): A literature review. J. Comput. Commun..

[46] Čolaković A., Hadžialić M. (2018). Internet of Things (IoT): A review of enabling technologies, challenges, and open research issues. Comput. Networks.

[47] Hashem I.A.T., Yaqoob I., Anuar N.B., Mokhtar S., Gani A., Khan S.U. (2015). The rise of “big data” on cloud computing: Review and open research issues. Inf. Syst..

[48] Dudin E., Smetanin Y.G. (2011). A review of cloud computing. Sci. Tech. Inf. Process..

[49] Krizhevsky A., Sutskever I., Hinton G.E. (2012). Imagenet classification with deep convolutional neural networks. Advances in Neural Information Processing Systems.

[50] Szegedy C., Ioffe S., Vanhoucke V., Alemi A.A. Inception-v4, inception-resnet and the impact of residual connections on learning. Proceedings of the 31st AAAI Conference on Artificial Intelligence.

[51] Vinyals O., Toshev A., Bengio S., Erhan D. Show and tell: A neural image caption generator. Proceedings of the IEEE Conference on Computer Vision and Pattern Recognition.

[52] Xu K., Ba J., Kiros R., Cho K., Courville A., Salakhudinov R., Zemel R., Bengio Y. Show, attend and tell: Neural image caption generation with visual attention. Proceedings of the 32nd International Conference on Machine Learning.

[53] Bahdanau D., Cho K., Bengio Y. (2014). Neural machine translation by jointly learning to align and translate. arXiv.

[54] Cho K., Van Merriënboer B., Gulcehre C., Bahdanau D., Bougares F., Schwenk H., Bengio Y. (2014). Learning phrase representations using RNN encoder-decoder for statistical machine translation. arXiv.

[55] Simonyan K., Zisserman A. (2014). Very deep convolutional networks for large-scale image recognition. arXiv.

[56] Deng J., Dong W., Socher R., Li L.J., Li K., Fei-Fei L. Imagenet: A large-scale hierarchical image database. Proceedings of the IEEE Conference on Computer Vision and Pattern Recognition.

[57] Parra L., Sendra S., Lloret J., Bosch I. (2015). Development of a conductivity sensor for monitoring groundwater resources to optimize water management in smart city environments. Sensors.

[58] Murphy K., Heery B., Sullivan T., Zhang D., Paludetti L., Lau K.T., Diamond D., Costa E., O’Connor N., Regan F. (2015). A low-cost autonomous optical sensor for water quality monitoring. Talanta.

[59] Obrovski B., Bajić J., Mihajlović I., Miloradov M.V., Batinić B., Živanov M. (2016). Colorimetric fiber optic probe for measurement of chemical parameters in surface water. Sens. Actuators B Chem..

[60] Parra L., Rocher J., Escrivá J., Lloret J. (2018). Design and development of low-cost smart turbidity sensor for water quality monitoring in fish farms. Aquacult. Eng..

[61] Heery B., Briciu-Burghina C., Zhang D., Duffy G., Brabazon D., O’Connor N., Regan F. (2016). ColiSense, today’s sample today: A rapid on-site detection of *β*-D-Glucuronidase activity in surface water as a surrogate for E. coli. Talanta.

[62] Donohoe A., Lacour G., McCluskey P., Diamond D., McCaul M. (2018). Development of a Cost-Effective Sensing Platform for Monitoring Phosphate in Natural Waters. Chemosensors.

[63] Allen M., Preis A., Iqbal M., Whittle A.J. (2012). Case study: A smart water grid in Singapore. Water Pract. Technol..

[64] Xie Z., Lou I., Ung W.K., Mok K.M. (2012). Freshwater algal bloom prediction by support vector machine in macau storage reservoirs. Math. Prob. Eng..

[65] Dong J., Wang G., Yan H., Xu J., Zhang X. (2015). A survey of smart water quality monitoring system. Environ. Sci. Pollut. Res. Int..

[66] Zhang D., O’Connor E., Sullivan T., McGuinness K., Regan F., O’Connor N.E. Smart multi-modal marine monitoring via visual analysis and data fusion. Proceedings of the 2nd ACM International Workshop on Multimedia Analysis for Ecological Data.

[67] Mac Cárthaigh M. (2005). Flooding in the Dodder Catchment 26 August 1986 (Hurricane Charlie) & 2 December 2003. www.epa.ie/pubs/reports/water/flows/EPAfloodingdodder2005.doc.

[68] Zhang D. (2015). A multi-Modal Smart Sensing Network for Marine Environmental Monitoring. Ph.D. Thesis.

